# Critical Serum Creatinine Values in Very Preterm Newborns

**DOI:** 10.1371/journal.pone.0084892

**Published:** 2013-12-30

**Authors:** Alexandra Bruel, Jean-Christophe Rozé, Cyril Flamant, Umberto Simeoni, Gwenaëlle Roussey-Kesler, Emma Allain-Launay

**Affiliations:** 1 Department of Pediatrics, Nantes University Hospital, University of Nantes, Nantes, France; 2 Department of Neonatalogy, Nantes University Hospital, University of Nantes, Nantes, France; 3 INSERM, CIC 004, Nantes University Hospital, Nantes, France; 4 INRA, Nantes University Hospital, UMR 1280 Physiologie des adaptations nutritionnelles, Nantes, France; 5 Department of Neonatalogy, AP-HM & Aix-Marseille University, Marseille, France; University of Western Australia, Australia

## Abstract

**Background:**

Renal failure in neonates is associated with an increased risk of mortality and morbidity. But critical values are not known.

**Objective:**

To define critical values for serum creatinine levels by gestational age in preterm infants, as a predictive factor for mortality and morbidity.

**Study Design:**

This was a retrospective study of all preterm infants born before 33 weeks of gestational age, hospitalized in Nantes University Hospital NICU between 2003 and 2009, with serum creatinine levels measured between postnatal days 3 to 30. Children were retrospectively randomized into either training or validation set. Critical creatinine values were defined within the training set as the 90^th^ percentile values of highest serum creatinine (HSCr) in infants with optimal neurodevelopmental at two years of age. The relationship between these critical creatinine values and neonatal mortality, and non-optimal neural development at two years, was then assessed in the validation set.

**Results and Conclusion:**

The analysis involved a total of 1,461 infants (gestational ages of 24-27 weeks (n=322), 28-29 weeks (n=336), and 30-32 weeks (803)), and 14,721 creatinine assessments. The critical values determined in the training set (n=485) were 1.6, 1.1 and 1.0 mg/dL for each gestational age group, respectively. In the validation set (n=976), a serum creatinine level above the critical value was significantly associated with neonatal mortality (Odds ratio: 8.55 (95% confidence interval: 4.23-17.28); p<0.01) after adjusting for known renal failure risk factors, and with non-optimal neurodevelopmental outcome at two years (odds ratio: 2.06 (95% confidence interval: 1.26-3.36); p=0.004) before adjustment. Creatinine values greater than 1.6, 1.1 and 1.0 mg/dL respectively at 24-27, 28-29, 30-32 weeks of gestation were associated with mortality before and after adjustment for risk factors, and with non-optimal neurodevelopmental outcome, before adjustment.

## Introduction

Assessing the impact of renal function on mortality and morbidity in preterm infants is challenging. Infants with high serum creatinine levels display an increased mortality rate, ranging from 11% to 70% [1-6]. However, this association with mortality or long-term neurodevelopment remains unclear, likely due to the lack of consensus regarding the definition of renal failure and/or the low statistical power of published studies. 

Reference values have been proposed for glomerular filtration rates according to gestational age (GA) [7-9] as well as for serum creatinine levels, which are more commonly used in clinical practice [8,10-12]. However, there is currently no consensus on the thresholds established for the diagnosis of acute renal failure. Some studies have proposed creatinine cut-off values of 1.1 mg/dL to 2 mg/dL after 48 hours, or up to 1.3 mg/dL after 60 hours of age [1,2,13]. Others have proposed definitions based on lack of the initial decrease in serum creatinine levels [14,15]; or conversely, on increased serum creatinine levels above the 99^th^ interval limit based on control studies [16]. More recently, Askenazy et al. proposed an increase in serum creatinine of 0.3 mg/dL, according to the definition of the AKIN (Acute Kidney Injury Network) [15-17].

Acute renal failure is known to be an independent predictive marker of morbidity and mortality in adults, as well as in patients from pediatric intensive care units [18,19]. Some studies on preterm infants have indicated that kidney disease directly affects mortality rates [4,6,17,20], but did not define serum creatinine cut-off values. The lack of an established definition of renal failure in preterm infants, and the direct impact of this complication on mortality and/or morbidity makes this an important area of investigation. The aim of this study was to determine the critical serum creatinine cut-off values associated with mortality and with a non-optimal neurodevelopmental outcome at two years of age and to determine if this association persists after adjustment for confounding variables. 

## Methods

### Data collection and study population

This is a retrospective cohort study of clinical and biological data recorded prospectively. Between January 2003 and December 2009, all preterm infants born before 33 weeks of gestation in Nantes University Hospital (France), and who had at least one or more serum creatinine measurements obtained between postnatal days 3 to 30, were included in this study. Infants were randomized proportionately in a 1:2 ratio into either a training set, used to define critical values, or a validation set. Children with genetic abnormalities or malformations were excluded from this study (cohort profile). Each set was subdivided according to GA (24-27, 28-29, and 30-32 weeks) to obtain homogeneous groups for the explanation of renal risk factors (ductus arteriosus, infections, surgery, etc.). Initial data related to the pregnancy, delivery, and medical care of these infants were prospectively recorded during hospitalization in the maternal and neonatal units and were then retrospectively collected and analyzed. At discharge, all surviving infants from the two groups were enrolled in the LIFT cohort (Loire Infants, regional Follow up Team) [21]. This cohort is registered at the French CNIL (Commission Nationale de l'Informatique et des Libertés, No. 851117). Approval of the use of the data for this study was obtained from the institutional review board of the University Hospital of Nantes, and parents gave written informed consent before infants were enrolled in follow-up.

### Neonatal characteristics

Due to the correlation between GA and birth weight, we used the weight Z-score index to assess the role of intrauterine growth. Z-scores were calculated via the LMS method [22], using Olsen’s growth chart for preterm infants [23]. Neonatal characteristics and renal risk factors already reported in literature [14], were noted for each patient. To treat cases of patent ductus arteriosus, we first used three doses of ibuprofen. When this treatment failed, surgery was discussed. Nosocomial infection was defined as an infection treated with antibiotics for >3 consecutive days (usually penicillin, glycopeptides, and aminoglycosides). Hemodynamic instability was considered if catecholamine treatment was administered. The severity of bronchodysplasia was defined according to the duration of oxygen therapy (<28 days, more than 28 days before 36 weeks of GA, or persistent oxygen therapy after 36 weeks of GA). Cranial ultrasonography was performed to detect intraventricular hemorrhage, and other lesions were diagnosed by Magnetic Resonance Imaging at term. In our study, cerebral lesions included stage III or IV intraventricular hemorrhage and cystic periventricular leukomalacia. 

### Biological assessment

For each infant, serum creatinine levels, sodium levels, and clinical data were prospectively recorded from hospital records using patient identification numbers. Serum creatinine was measured using a compensated Jaffe’s kinetic method, and levels obtained between days 3 and 30 were recorded (the day of birth was considered day zero). As described by Baraton et al., maximal natremia variations occur around day 15 in children born before 28 weeks of gestation; we thus collected all of the biological data between day 3 and 30 [24]. Data after day 30 were not taken into account because of the low frequency of renal injury after the first month of life in premature infants. The highest serum creatinine (HSCr) values, as well as the highest and lowest serum sodium values, were recorded. Changes in sodium levels were defined as the difference between the highest and lowest measured values during the first month of life [24].

### Neurodevelopmental outcome

Neurodevelopmental assessment involved evaluation of neuromotor function by a pediatrician and of psychomotor development by a specialized psychologist. Two-year neuromotor function was considered non-optimal when severe or moderate neuromotor impairment was noted, resulting in a diagnosis of cerebral palsy; or when clinical examination revealed milder neurological signs of abnormal movement during independent walking (phasic stretch of the triceps surae muscle and imbalance of passive axial tone, with predominance of extensor tone). A psychomotor developmental quotient (DQ) was calculated after assessment using the revised Brunet-Lezine test [25], with a score <85 indicating non-optimal psychomotor development. Also, in cases where neurological impairment was too severe for the child to take the test, he/she was included in the non-optimal psychomotor development group. In addition, if a psychological evaluation could not be performed, psychomotor outcome was determined with the Ages & Stages Questionnaire, a parent-completed developmental assessment tool validated through comparison with the revised Brunet-Lezine test [26]. In this case, non-optimal neurodevelopment was defined as an overall score ≤ 185. Patients with non-optimal neuromotor function and/or psychomotor development were considered to have a non-optimal neurodevelopmental outcome.

### Statistical analysis

Definition of critical values in the training group: To determine our critical values, we considered the highest serum creatinine values of children from the training group showing an optimal neurodevelopmental outcome at two years of corrected age. The 90^th^ percentile of HSCr levels from these children with an optimal neurodevelopmental outcome were then used to define critical values for each GA subgroup, as previously reported for other parameters like blood pressure [27].

 Evaluation of critical values in the validation group: The established critical creatinine values were then validated in the validation group. Khi^2^ and Fisher’s exact tests were used to test the association between neonatal characteristics (including previously reported risk factors for renal failure) [28] and serum creatinine levels exceeding critical values. Since changes in serum sodium levels affect neurodevelopmental outcome, the variations in serum sodium levels between infants with HSCr values greater than or less than the critical cut-off value were studied [24]. The association between changes in serum sodium and outcome was also assessed by logistic regression. Finally, the association between an HSCr above the critical value and mortality was evaluated by logistic regression. This analysis was performed before and after adjustment for the following confounding risk factors: GA, weight Z-score, ductus arteriosus, catecholamine treatment, nosocomial infection, bronchodysplasia, cerebral lesions, necrotizing enterocolitis, neonatal surgery, change in serum sodium levels. All analyses were performed with SPSS 15.0. 

## Results

### Study population characteristics

**Figure 1:**
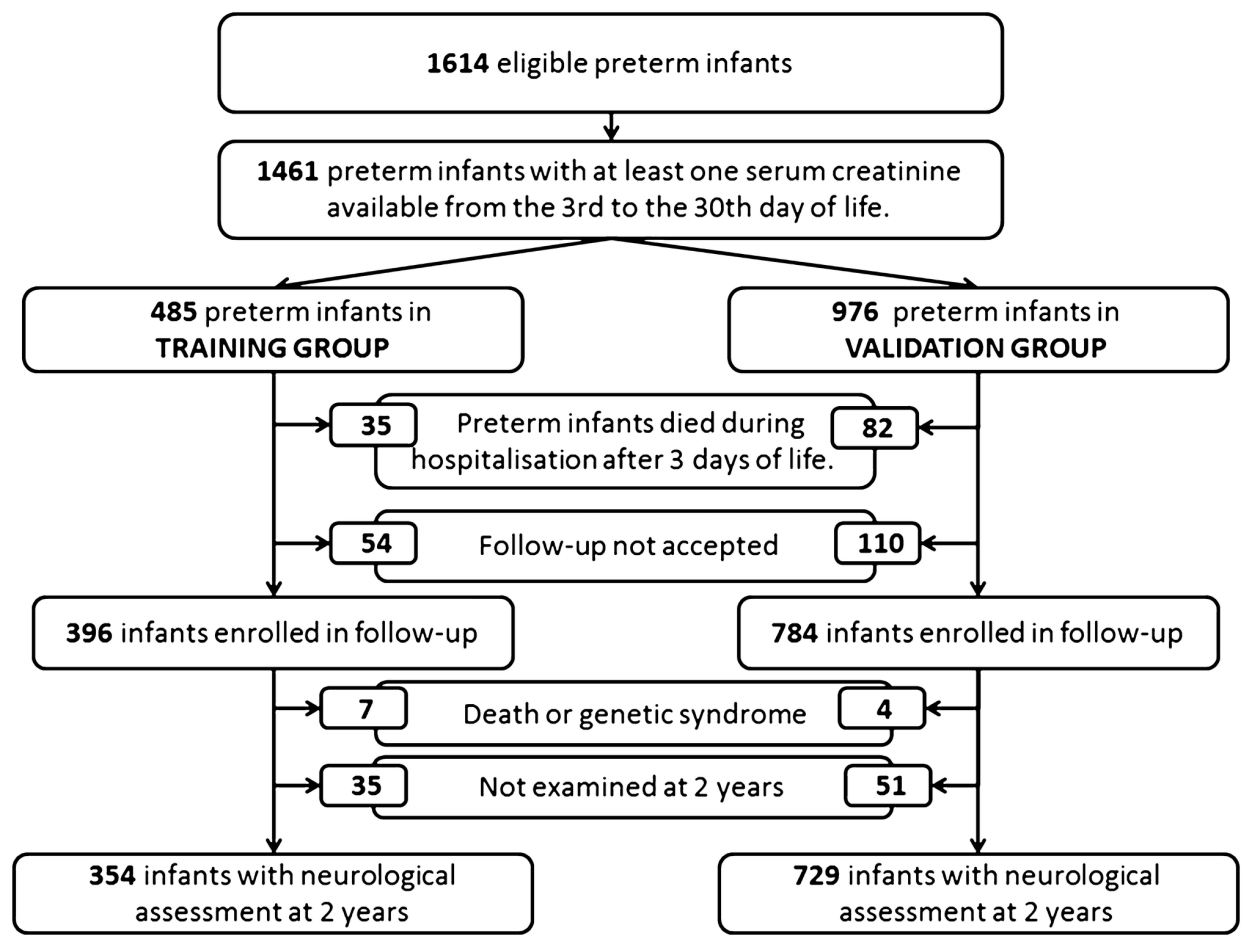
Cohort profile.

The cohort profile is summarized in Figure 1. During the seven-year period analyzed, 1,461 out of the 1,614 eligible preterm infants born at < 33 weeks of GA had at least one serum creatinine value measured between days 3 to 30, and were included in the study. The median number of creatinine assessments per child was six (quartiles: 3-12), with a total of 14,930 measurements. These 1,461 infants were allocated into either the training (n=485), or the validation set (n=976). Neonatal characteristics and outcomes are presented in Table 1. Apart from cerebral lesions, no significant difference was found between the two sets.

**Table 1:** Characteristics of children in the training and validation groups.

		**Training group**		**Validation group**		**p value**
		**n= 485**	**%**	**n= 976**	%	
**Neonatal characteristics**						
Gestational age (weeks)						
	24-27 weeks	102	(21	220	22	0.57
	28-29 weeks	107	(22	229	24	
	30-32 weeks	276	(57	527	54	
Birth weight Z-score						
	<-1 SD	96	(20)	217	(22)	0.41
	(-1 ; 0) SD	204	(42)	477	(43)	
	> 0 SD	185	(38)	342	(35)	
Female		215	(44)	439	(45)	0.83
Mean creatinine ( mg/dl)						
	24-27 weeks	1.4	(0.7)	1.4	(0.7)	0.27
	28-29 weeks	0.8	(0.4)	0.8	(0.3)	
	30-32 weeks	0.7	(0.4)	0.7	(0.3)	
Number of creatinine assessment						
	24-27 weeks	20	(17)	22	(18)	0.78
	28-29 weeks	11	(10)	11	(9)	
	30-32 weeks	5	(6)	6	(7)	
Sodium variation (meq/l)						
	24-27 weeks	21	(11)	20	(10)	0.98
	28-29 weeks	14	(10)	14	(7)	
	30-32 weeks	9	(6)	9	(6)	
Patent ductus arteriosus		59	(12)	119	(12)	0.98
Catecholamine treatment		42	(9)	70	(7)	0.32
Nosocomial infection		123	(25)	280	(29)	0.18
Bronchodysplasia: oxygenotherapy						
	No oxygen	269	(56)	491	(50)	0.09
	< 28 days	131	(27)	323	(33)	
	28 days- 36 weeks of GA	58	(12)	118	(12)	
	> 36 weeks of GA	27	(6)	44	(5)	
Cerebral lesions		17	(4)	64	(7)	0.02
Neonatal surgery		46	(10)	86	(9)	0.67
Necrotizing enterocolitis		11	(2)	30	(3)	0.38
**Neonatal outcome**						
	Death	35	(7)	82	(8)	0.43
	Alive	450	(93)	894	(92)	
**Outcome at 2 years**		**n= 396**	**(%)**	**n= 784**	(%)	
	Optimal outcome	269	(68)	550	(70)	0.31
	Non-optimal outcome	85	(22)	179	(23)	
	Missing data	35	(9)	51	(7)	
	Genetic anomalies	6	(2)	2	(3)	
	death	1	(3)	2	(3)	

### Definition of critical HSCr values in the training set

The mean HSCr values for the GA subgroups were 1.4 mg/dL at 24-27 weeks, 0.8 mg/dL at 28-29 weeks, and 0.7 mg/dL at 30-32 weeks (Table 1). Critical values were determined by analysis of the 269 children in the training set, who showed optimal neurodevelopmental outcome at two years of corrected age. The 90^th^ percentile HSCr values from this population were used to define the critical values for each GA subgroup, which were found to be 1.6 mg/dL at 24-27 weeks, 1.1 mg/dL at 28-29 weeks, and 1.0 mg/dL at 30-32 weeks. 

### Critical HSCr values in the validation set

Among the 976 children included in the validation group, 143 had an HSCr above the critical value corresponding to their GA (14.7%). Furthermore, for infants that displayed HSCr values in the critical range, we observed that peak values were reached significantly (p<0.01) later for the 24-27 weeks GA group (median: day 11; quartiles: 6-16), compared to 28-29 weeks (median: day 5; quartiles: 3-11), or 30-32 weeks (median: day 3; quartiles: 3-5).

### Neonatal risk factors for having an HSCr above the critical value

Having an HSCr above the critical value was significantly associated with specific neonatal characteristics, such as low GA and low birth weight Z-score. It was also associated with risk factors for renal failure, such as infectious complications, hemodynamic instability, iatrogenic interventions, and other comorbidities associated with prematurity (Table 2). 

**Table 2:** Neonatal risk factors for highest serum creatinine > critical values (1.6 mg/dL at 24-27 weeks, 1.1 mg/dL at 28-29 weeks, and 1.0 mg/dL at 30-32 weeks).

**Risk factors**		**Adjusted OR**	**95%CI**		**p**
Gestational age (weeks)	30-32 weeks	1.00			
	28-29 weeks	1.40	(0.87 ; 2.25)		0.16
	24-27 weeks	3.27	(2.17 ; 4.94)		0.01
Birht weight Z-score	> 0 SD	1.00			
	(-1 ; 0) SD	0.76	(0.50 ; 1.14)		0.18
	<- 1 SD	1.00	(0.63 ; 1.59)		0.98
Female		0.86	(0.60 ; 1.24)		0.43
Multiple pregnancy		0.67	(0.44 ; 1.02)		0.06
Antenatal corticotherapy		0.72	(0.49 ; 1.03)		0.07
Natremia variation (by 10 meq/l)		2.3	(1.89 ; 2.79)	0.01	
Patent ductus arteriosus		3.70	(2.40 ; 5.71)		0.01
Catecholamine treatment		11.15	(6.63 ; 18.71)		0.01
Nosocomial infection		2.65	(1.47 ; 3.82)		0.01
Bronchodysplasia: oxygenotherapy duration	No oxygen	1.00			
	< 28 days	1.73	(1.13 ; 2.65)		0.01
	28 days- 36 weeks of GA	2.54	(1.49 ; 4.33)		0.01
	> 36 weeks of GA	8.62	(4.44 ; 16.75)		0.01
Cerebral lesions		3.99	(2,33 ; 6.87)		0.01
Neonatal surgery		3.25	(1.99 ; 5.32)		0.01
Necrotizing enterocolitis		1.81	(0.76 ; 4.30 )		0.18

### Interaction between changes in serum sodium levels and having an HSCr above the critical value

Change in serum sodium, as well as minimal and maximal serum sodium levels are presented in Table 3. Variation in serum sodium was significantly more important for children with an HSCr value above the critical value, for the three GA groups, and serum sodium change decreased with GA (Table 3). Serum sodium change (by 10 meq/l) was associated with having an HSCr above the critical value (odds ratio (OR)=2.3; 95% confidence interval (CI): 1.89-2.79; p=0.01), and with neurodevelopmental outcome at two years (OR=1.4; 95%: CI: 1.1-1.8; p=0.06).

**Table 3:** Natremia according to highest serum creatinine value (HSCr).

Groups of preterm infants according to GA and HSCr		N	Age at time of max. natremia		Max. serum sodium level		Age at time of min. natremia		Min. serum sodium level		Change in serum sodium level	
			mean (days)	p	mean (mmol/l)	p	mean, days	p	mean (mmol/l)	p	mean (mmol/l)	p
**24-27 weeks**												
	HSCr > critical value	59	11.6	0.81	149.5	0.01	15.5	0.97	124.1	0.01	25.5	0.01
	HSCr < critical value	161	11.2		145.6		15.3		127.7		17.9	
**28-29 weeks**												
	HSCr > critical value	31	12.4	0.02	145.1	0.02	9.4	0.60	125.4	0.01	19.7	0.01
	HSCr < critical value	198	6.4		143.2		8.4		130.5		12.7	
**30-32 weeks**												
	HSCr > critical value	53	6.4	0.76	143.5	0.01	4.5	0.30	131.7	0.02	11.7	0.01
	HSCr < critical value	474	6.9		141.8		6.0		133.3		8.5	

Critical values: 1.6 mg/dL at 24-27 weeks, 1.1 mg/dL at 28-29 weeks, and 1.0 mg/dL at 30-32 weeks

### Association between critical HSCr values and outcome in the validation group

**Figure 2:**
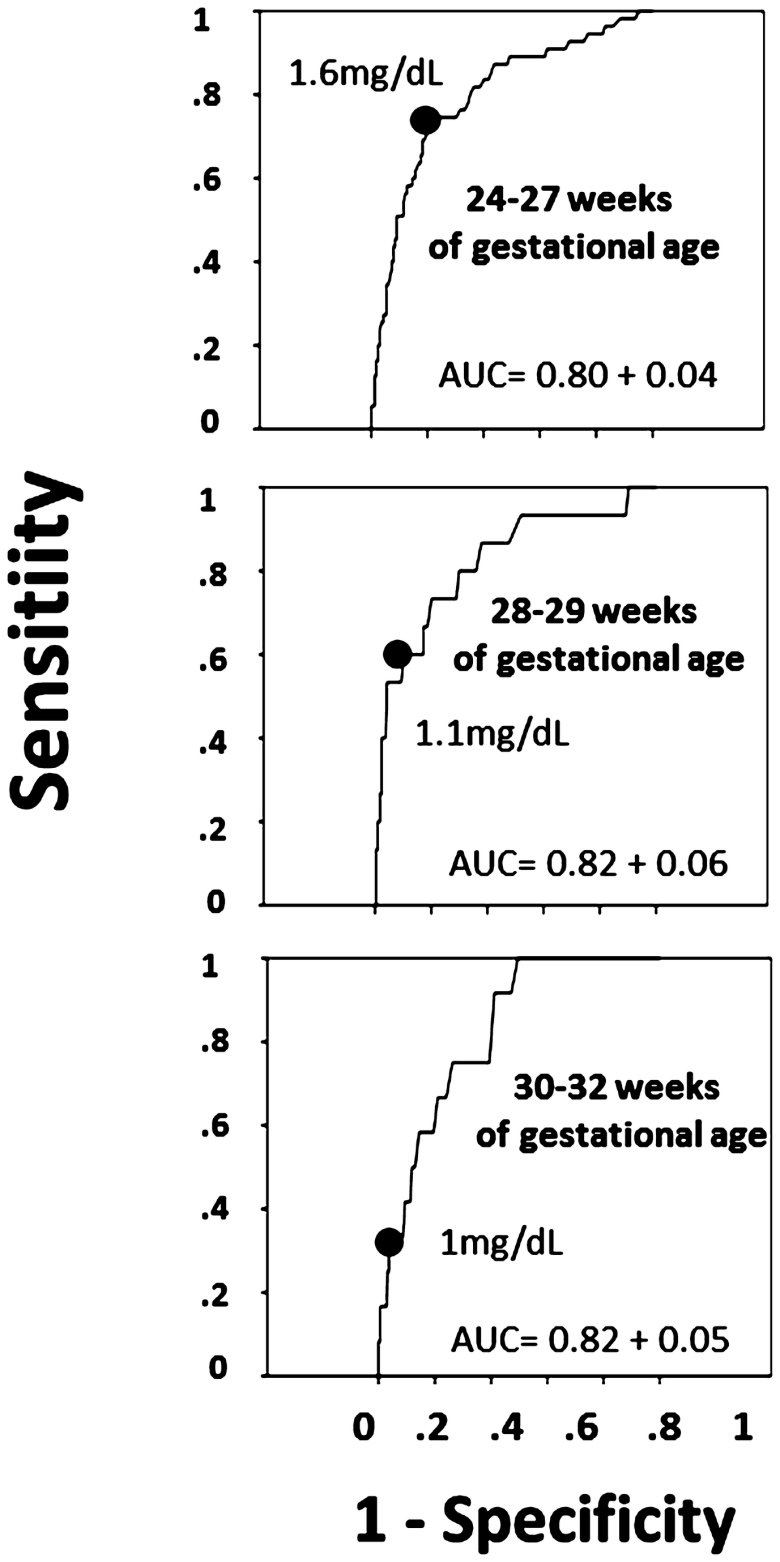
Critical values of creatinine (indicated by black circle) and receiver operating curve for creatinine to predict mortality, according to gestational age, in validation set.

Critical HSCr values and receiver operating curves for serum creatinine according to gestational age are represented on Figure 2. Outcome of children according to their HSCr is presented in Table 4. After multivariate analysis, we observed an association between having an HSCr above critical values and mortality (adjusted OR=11.16; 95% CI: 6.86-18.15; p<0.01), which remained strongly positive after adjusting for GA, Z-score, and neonatal risk factors for renal impairment (adjusted OR= 8.55; 95% CI: 4.23-17.28; p<0.01). An HSCr superior to the critical value was also associated with non-optimal neurodevelopment (OR=2.06; 95% CI:1.26-3.36; p<0.01); however, after adjusting for confounding variables (except sodium variation), this association tended to remain significant (adjusted OR=1.68; CI: 0.97; 2.92; p=0.063). After adjustment for sodium variation, this association was no longer significant (aOR=1.48; 95% CI: 0.84-2.61; p=0.17). 

**Table 4:** Association between renal impairment and death or non-optimal neurodevelopmental outcomes in the validation set.

	**Odds ratio**	**95% CI**	**p-value**	
**HSCr > critical values as risk factor for death (n=976)**				
No adjustment	11.2	(6.9; 18.2)		0.001
Adjustment for GA, birth weight Z-score and sex	8.6	(5.0 ; 14.6)		0.001
Adjustment for GA, birth weight Z-score, sex and risk factors for HSCr > critical values	9.3	(4.7 ; 18.7)		0.001
Adjustment for GA, birth weight Z-score, sex, risk factors for HSCr > critical values and diuretic treatment	9.5	(4.7 ; 19.1)		0.001
Adjustment for GA, birth weight Z-score, sex, risk factors for HSCr > critical values, diuretic treatment and delta natremia	8.8	(4.3 ; 17.8)		0.001
**HSCr> critical values as risk factor for Non optimal Outcome (n=729)**				
No adjustment	2.1	(1.3 ; 3.4)		0.004
Adjustment for GA, birth weight Z-score and sex	1.9	(1.2 ; 3.1)		0.013
Adjustment for GA, birth weight Z-score, sex and risk factors for HSCr > critical values	1.7	(0.97 ; 2.9)		0.063
Adjustment for GA, birth weight Z-score, sex, risk factors for HSCr > critical values and diuretic treatment,	1.7	(0.97 ; 2.9)		0.062
Adjustment for GA, birth weight Z-score, sex, risk factors for HSCr > critical values, diuretic treatment and delta natremia	1.5	(0.85 ; 2.6)		0.16

Abbreviations: HSCr, highest serum creatinineCritical values: 1.6 mg/dL at 24-27 weeks, 1.1 mg/dL at 28-29 weeks, and 1.0 mg/dL at 30-32 weeks)

## Discussion

Preterm infants with an HSCr above 1.6 mg/dL at 24-27 weeks, 1.1 mg/dL at 28-29 weeks, and 1.0 mg/dL at 30-32 weeks, had higher risk of mortality and non-optimal neurodevelopmental at two years. Thus, we propose to use these critical values to identify preterm infants at high risk of renal failure. 

In the literature, it is difficult to find a consensus regarding the definition of renal impairment in preterm infants. Therefore, in the present study, we have defined critical values for serum creatinine, a marker of renal function. Critical values were described in the 1970's [29] as an indication for healthcare providers of potentially life-threatening laboratory results immediately after testing. Moreover, the most appropriate approach for defining a critical value seems to be based on the patient’s outcome [30]. Here, the 90^th^ percentile of HSCr values (from postnatal days 3-30) of children with an optimal two-year outcome were used to determine the serum creatinine critical values, which were then validated in a second group with the same neonatal characteristics. Thus, limits arising from intrinsic characteristics of the deceased infants within our cohort were overcome through internal validation of our critical values [27]. Importantly, risk factors for HSCr above the critical values were similar to risk factors for renal failure described in the literature [1,4,10]. 

Additionally, we observed that GA had an impact on renal function. In fact, the critical HSCr values of 1.6, 1.1 and 1.0 mg/dL decreased with GA (24-27 weeks, 28-29 weeks, 30-32 weeks, respectively). Other cohort studies have noted similar evolutions of serum creatinine levels over time [8,10-12], with peak values reported in the first days of life, followed by a progressive decrease. Interestingly, in preterm infants displaying serum creatinine values above the critical values, the postnatal age for HSCr varied as a function of GA. We found that infants of 24-27 weeks of GA reached their peak HSCr value significantly later (day 11) than those born at 28-29 weeks (day 5) and 30-32 weeks (day 3). This could be explained by patent ductus arteriosus, sepsis, necrotizing enterocolitis, and the iatrogenic interventions used for treating these complications, which frequently occur in this population. Thus, renal impairment could be an indicator of the severity of neonatal disorders, especially in younger infants.

Also, we observed that the critical values determined here were significantly associated with neonatal mortality. Studies on renal failure have reported high mortality in preterm infants [1,4,13,17], but none of these studies proposed a critical value or a definition for renal failure. In the absence of comparative analysis, Walker et al. suggested a median creatinine value of 1.1 mg/dL (0.6-1.7, 10^th^-90^th^ percentile) in preterm infants of 28 weeks of GA, who had died, and 0.4 mg/dL (0.2-0.7) for those who had been discharged [4]. Our results are in agreement with those of Askenazi et al [17]. Another study attempted to demonstrate the impact of acute kidney injury on survival in very low birth weight infants; however, after adjusting for confounding variables, renal injury was not associated with mortality. This was probably due to the lack of statistical power, or to a difficulty in defining acute kidney injury [6]. It is clear from these studies that a consensus definition for renal failure is necessary for demonstrating its independent effect on mortality and morbidity, as has already been shown in cohorts of adults (RIFLE score, AKIN classification) and children (pediatric RIFLE) in intensive care units [18-20]. However, even in adult or pediatric cohorts, the lack of a consensus on a definition for acute renal failure has resulted in a limitation of each published score to predict outcome with a strong sensibility [31]. The increase in serum creatinine value is a marker of the severity of the illness in preterm newborns, strongly associated with mortality. Here, we focused on proposing critical values for serum creatinine, which have predictive power for patient outcome, providing clinicians with a method to detect children at high risk of mortality and allowing them to take the appropriate measures. Serum creatinine value is an interesting new element in neonatal severity scores, particularly useful in the subgroup 30-32 weeks of gestational age, when other signs of severity (neurological, respiratory) are less frequent. These findings could be a first step towards the establishment of an agreed definition for renal failure. 

Moreover, critical HSCr values are significantly associated with neurodevelopmental outcome at two years, before and after adjustment for GA, birth weight Z-Score, and gender. However, after adjusting for confounding variables (except for changes in sodium), we found the association to be at the limit of significance. Moreover, after adjusting for sodium changes, the association was no longer significant, suggesting that changes in serum sodium are the main factor impacting outcome in very premature newborns. Additionally, changes in serum sodium levels are significantly associated with having HSCr above critical values, and with outcome at two years, and interfere with the relationship between critical HSCr levels and neurodevelopmental outcome. Having an HSCr above the critical value probably directly impacts on neonatal mortality, whereas its association with neurodevelopmental outcome is probably a result of variations in sodium levels. Taken together, we confirm changes in sodium as a marker of severity of illness in very preterm infants, which is associated with neurodevelopmental outcome [24]. 

This study has some limitations, including the method used for measuring serum creatinine levels, which has the potential for introducing bias due to inter-laboratory technical variability [32]. We used a modified method based on Jaffe’s kinetic. Although our dosage kit is standardized with isotopic dilution mass spectrometry and validated as having minimal interference with chromogens such as bilirubin, this is not the gold standard method and recommendations for best practices have proposed the use of an enzymatic assay for analysis of serum creatinine in children [33,34]. Our method could therefore under-evaluate serum creatinine levels, as bilirubin and hemoglobin levels are often high in preterm infants. Thus, each testing laboratory should cautiously interpret our critical values, which must be validated with enzymatic assays. Moreover, the major studies investigating the associating between acute kidney injury (AKI) in preterm infants and mortality, have used the Jaffe method [6], which is known to be unreliable for lower values [35]. This could be a limitation in using these definitions of AKI based on an increase of serum creatinine from the previous value. Nevertheless, serum creatinine is the most commonly and easily used marker of renal function. In our cohort, one bias could be due to the retrospective selection of children by their laboratory results, as we only studied children whose clinician had ordered a serum creatinine test. Of the 1,614 children studied, 153 had not had their creatinine levels measured. The aim of our study was to define critical values of serum creatinine so they could be utilized by clinicians as a new tool for the management of high-risk preterm infants. 

This study did not investigate the 153 children who had not had their creatinine levels measured, as these patients were assessed as having a low-risk outcome. This retrospective study analyzed a very large cohort of 1,461 infants. Critical values were determined on 14,721 creatinine measurements, making our study unique despite its limitations. Furthermore, the retrospective analysis of prospectively collected data avoids the bias that is due to clinician appreciation, inevitable in some prospective studies. Therefore, while validation of these data in a larger multi-site cohort study will be necessary to reduce bias, prior development of a uniform, standardized enzymatic method for measuring creatinine levels would be beneficial.

## Conclusion

This study provides new insights into the association between renal impairment in very preterm newborns and mortality or morbidity. The calculation of critical serum creatinine values according to GA will be helpful in clinical practice and research, but these critical values must now be tested and validated in other cohorts of preterm infants. 

## Supporting Information

Table S1 **Characteristics of children with optimal neurodevelopmental outcome in the training and validation groups.**
(DOCX)
