# Supplementary material for: Critical Serum Creatinine Values in Very Preterm Newborns
Source: PLoS One. 2013 Dec 30;8(12):e84892. doi: 10.1371/journal.pone.0084892 (PMC3875547; doi:10.1371/journal.pone.0084892)
Supplement: Table S1 — Characteristics of children with optimal neurodevelopmental outcome in the training and validation groups. (DOCX) [file pone.0084892.s001.docx]

**Supplementary table 1**: Characteristics of children with optimal neurodevelopmental outcome in the training and validation groups

|  |  | **Training group**  **n= 269** | | | |  | **Validation group**  **n= 550** | | | | **p value** |
| --- | --- | --- | --- | --- | --- | --- | --- | --- | --- | --- | --- |
| GA (weeks) | 24-27 w | 40 | ( | 15 | ) |  | 90 | ( | 16 | ) | 0.79 |
|  | 28-29 w | 69 | ( | 26 | ) |  | 132 | ( | 24 | ) |  |
|  | 30-32 w | 160 | ( | 60 | ) |  | 328 | ( | 60 | ) |  |
| Weight Z-score | <-1 SD | 54 | ( | 20 | ) |  | 124 | ( | 23 | ) | 0.43 |
|  | [-1 ; 0] SD | 109 | ( | 41 | ) |  | 234 | ( | 43 | ) |  |
|  | > 0 SD | 106 | ( | 39 | ) |  | 192 | ( | 35 | ) |  |
| Female |  | 129 | ( | 48 | ) |  | 250 | ( | 46 | ) | 0.50 |
| Max. creatinine ( mg/dl) | 24-27 w | 1.09 | ( | 0.39 | ) |  | 1.09 | ( | 0.46 | ) | 0.58 |
|  | 28-29 w | 0.80 | ( | 0.24 | ) |  | 0.82 | ( | 0.36 | ) |  |
|  | 30-32 w | 0.73 | ( | 0.34 | ) |  | 0.67 | ( | 0.23 | ) |  |
| Number of creatinine assessment | 24-27 w | 17.2 | ( | 7.7 | ) |  | 17.1 | ( | 8.0 | ) | 0.21 |
|  | 28-29 w | 13.7 | ( | 9.3 | ) |  | 12.8 | ( | 5.5 | ) |  |
|  | 30-32 w | 8.3 | ( | 5.1 | ) |  | 8.8 | ( | 6.4 | ) |  |
| Sodium variation (meq/l) | 24-27 w | 21.3 | ( | 11 | ) |  | 20.0 | ( | 10 | ) | 0.80 |
|  | 28-29 w | 14.1 | ( | 10 | ) |  | 13.7 | ( | 7 | ) |  |
|  | 30-32 w | 8.6 | ( | 6 | ) |  | 8.8 | ( | 6 | ) |  |
| Patent ductus arteriosus |  | 25 | ( | 9 | ) |  | 43 | ( | 8 | ) | 0.47 |
| Catecholamine treatment |  | 10 | ( | 4 | ) |  | 17 | ( | 3 | ) | 0.64 |
| Nosocomial infection |  | 59 | ( | 22 | ) |  | 142 | ( | 26 | ) | 0.23 |
| Bronchodysplasia: oxygenotherapy duration | no oxygen | 155 | ( | 58 | ) |  | 281 | ( | 51 | ) | 0.15 |
|  | < 28 days | 73 | ( | 27 | ) |  | 193 | ( | 35 | ) |  |
|  | 28 days- 36 weeks of GA | 29 | ( | 11 | ) |  | 56 | ( | 10 | ) |  |
|  | > 36 weeks of GA | 12 | ( | 5 | ) |  | 20 | ( | 4 | ) |  |
| Cerebral lesions |  | 3 | ( | 1 | ) |  | 9 | ( | 2 | ) | 0.56 |
| Neonatal surgery |  | 20 | ( | 7 | ) |  | 36 | ( | 7 | ) | 0.64 |
| Necrotizing enterocolitis |  | 4 | ( | 2 | ) |  | 7 | ( | 1 | ) | 0.80 |
